# Early Social Experience Predicts Referential Communicative Adjustments in Five-Year-Old Children

**DOI:** 10.1371/journal.pone.0072667

**Published:** 2013-08-29

**Authors:** Arjen Stolk, Sabine Hunnius, Harold Bekkering, Ivan Toni

**Affiliations:** Donders Institute for Brain, Cognition and Behaviour, Radboud University Nijmegen, Nijmegen, The Netherlands; University of Tuebingen Medical School, Germany

## Abstract

A large body of work has focused on children’s ability to attribute mental states to other people, and whether these abilities are influenced by the extent and nature of children’s social interactions. However, it remains largely unknown which developmental factors shape children’s ability to influence the mental states of others. Building on the suggestion that collaborative experiences early in life might be crucial for the emergence of mental coordination abilities, here we assess the relative contribution of social exposure to familial and non-familial agents on children’s communicative adjustments to their mental model of an addressee (‘audience design’). During an online interactive game, five-year-olds spontaneously organized their non-verbal communicative behaviors according to their beliefs about an interlocutor. The magnitude of these communicative adjustments was predicted by the time spent at daycare, from birth until four years of age, over and above effects of familial social environment. These results suggest that the degree of non-familial social interaction early in life modulates the influence that children’s beliefs have on their referential communicative behavior.

## Introduction

Humans often use un-observable variables like beliefs, desires, and intentions to disambiguate agents’ behavior, attributing mental states to other people and to oneself [1], [2]. These mentalizing abilities emerge during early childhood [3] and variations in mentalizing skills appear to be related to social environmental factors [4]. Among these factors, collaborative experiences of a child with adult group members might play a crucial role [5], [6]. These interactions might allow children to gradually construct knowledge of the world, as well as knowledge of other people’s mental states, by capturing cognitive regularities that cooperative agents try to make transparent to the child [7]. Eventually, children start using this knowledge to manipulate the mental states of other agents during referential communicative interactions. For instance, 4-year-old children use presumed knowledge of an interlocutor to select linguistic behaviors designed to change those mental states, producing more explicit descriptions of a toy when speaking to a blind as compared to a non-blind addressee [8], and simpler utterances towards a toddler than an adult [9]. Five-year-old children can produce verbal requests that take into account the presumed knowledge of their interlocutor [10]. However, it remains largely unknown how children learn to adjust their referential communicative behaviors to their mental model of an addressee.

Here we elaborate on the suggestion that the extent and nature of the social interaction children experience will influence the development of children’s social understanding [5], [7], [11], [12], [13]. Humans are exceptional among existing hominids for experiencing early developmental exposure to cooperative nonkin [14], i.e. conspecifics that lack a genetic reason for collaborating, and it has been suggested that this developmental feature might boost motivational predispositions to share mental states with others [6]. We quantify one aspect of this faculty through audience design, i.e. adjustments of communicative acts to the presumed abilities and knowledge of an interlocutor [15]. Given that audience design presupposes control of the ability to share mental states with others, we focus on five-year-old children, i.e. children with fully-fledged theory of mind capacities [16]. We quantify developmental exposure to two main sources of social interactions experienced by children between zero and four years of age, namely familial and non-familial experiences. The former were quantified in terms of years of experience with siblings, and parents’ level of education. The latter were quantified in terms of days per week of attendance to daycare [11], [13], [17], [18], [19].

Audience design effects were quantified in a controlled experimental setting involving the production of referential non-verbal behaviors with a communicative goal [20], exploiting a protocol previously validated in adults [21]. In contrast to linguistic communication, the communicative behaviors evoked under these experimental conditions could not be directly based on previous concrete experiences. Accordingly, the novel communicative situation experienced by the children in this study allowed us to directly tap into their ability to influence the mental states of others through behaviors generated ex-novo. Five-year-old participants were told they were playing an online interactive game with a 2-year-old toddler and with a same-age peer, in alternation. In fact, a confederate performed the role of both addressees, while remaining blind to which one of the two roles he was performing in any given trial. Accordingly, both performance and response times of the two presumed addressees were matched. This feature of the protocol allowed us to test whether the mere belief that the child is communicating with addressees of different ability induces internally generated adjustments in the child behavior, over and above performance-related mutual adjustments [22], [23]. Furthermore, the precise quantification of children behavior afforded by this protocol distinguished between belief-driven adjustments restricted to the communicative components of the actions, and generic priming effects [24], [25]. These procedures allowed us to test whether the social environment experienced by a child early during his development influences his ability to adjust a self-generated communicative behavior to his mental model of the addressee.

## Materials and Methods

### Participants

The experiment was approved by the local medical ethical committee (ECG, Nijmegen, The Netherlands). Parents with 5-year-old children (N = 24, 12 females, mean age 5.09, range 5.02–5.16) were recruited from a database of the Baby Research Center Nijmegen. The children’s parents provided written informed consent for participation of their children in the study, and all participants received a book or monetary compensation for their visit.

### Experimental Design

**Figure 1:**
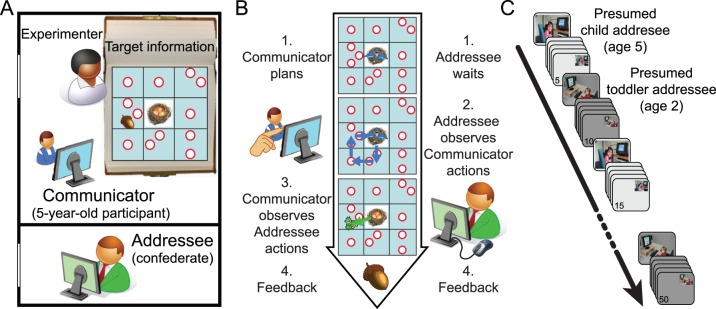
Task setup. (A) The Communicator, a 5-year-old participant, sat next to an Experimenter who provided the task instructions and the trial-specific location of the acorn but played no part in the communicative game. The Addressee, a confederate who performed the role of a toddler and a child (see panel C), while remaining blind to which one of the two roles he was performing in any given trial, sat outside the experimental room facing another monitor. (B) Each single trial encompassed four successive events. (1) the Experimenter showed to the Communicator only the location of the acorn (see panel A), and the Communicator had unlimited time to plan the movements; (2) the Communicator moved the bird icon on the game board by touching a touch-screen with a finger (the movements of the bird were visible to both Communicator and Addressee); (3) the Addressee moved the squirrel icon on the game board with a digital mouse (the movements of the squirrel were visible to both Communicator and Addressee); (4) both players received common feedback on the communicative success of the trial. Note that the bird, unlike the squirrel which could move freely, could only move to the center of each of the nine grid squares, and only through vertical or horizontal displacements. This feature of the task made it difficult for the Communicator and the Addressee to discriminate the location of multiple potential targets within a square (the white circles) on the basis of the location of the bird alone. (C) A digital photograph of the current presumed addressee was presented to the Communicator in full screen before the onset of each block of 5 trials, and in the top right corner during each block.

The game involves a Communicator (a 5-year-old participant, displayed as a bird on the game board) and an Addressee (the confederate, displayed as a squirrel) interacting on a digital game board with a 3×3 grid layout (see Figure 1A). On each trial, their joint goal was for the Addressee to collect an acorn from the game board. Given that knowledge of the acorn’s location in the game board was available to the Communicator only (on a printed copy of the game board, visible throughout the trial, see Figure 1A), a successful trial of this game required the Communicator to inform the Addressee where the acorn was located. Given the experimental setup, the Communicator could inform the Addressee only by moving the bird across the game board (event 2 in Figure 1B). The Addressee could then move the squirrel to the acorn’s location only by interpreting the meaning of the Communicator’s movements on the game board (event 3 in Figure 1B). For details on the experimental procedure see the Supplemental Material.

By touching a square on the screen with his/her finger, the Communicator could move the bird token to that square, and this movement was also visible to the Addressee. The bird could only move to the center of each of the nine grid squares, and only through vertical or horizontal displacements. This feature of the task was introduced to create a spatial disparity between the movements of the bird and the potential locations of the target object (any of the thirteen white circles, see Figure 1B). Namely, the bird could not be overlaid on the precise location of the acorn when a square contained more than one white circle (see Manipulation of task difficulty of the Supplemental Material for details). The Communicator had no restrictions on planning time (event 1 in Figure 1B) or on movement time (event 2). The end of the movement epoch was marked by the return of the bird on the central square of the game board (nest). At this point, the token of the Addressee (the squirrel) appeared, in the center of the digital game board, visible to both players. The Addressee moved the squirrel to the location deemed appropriate given the movements of the Communicator (event 3). The Addressee had no temporal or spatial restrictions on the movements of the squirrel on the game board. Successful trials, in which the Addressee had moved to the location of the target, resulted in the presentation of a large acorn on the screen (event 4). A red “no” icon was presented over a small acorn for unsuccessful trials.

There were a total of 50 trials, subdivided in blocks of five trials (∼35 min, Figure 1C). Each child was informed that he would be playing an interactive game with two addressees in turns; either a toddler (‘2-year-old’) or a same–age peer (‘5-year-old’). They were told that the game partners were sitting in other rooms and that they could see the bird token and the digital game board on their monitors. There were two pairs of fictitious child-toddler addressees, two presentation orders of child-toddler addressees, and two sets of target configurations, counterbalanced over participants.

### Quantification of the Social Environment

Given that the extent and nature of the social interactions experienced by children is widely thought to influence the development of their social understanding [5], [7], [12], [13], we considered two main sources of social interactions experienced by children, namely familial and non-familial experiences, reconstructed from interviews with the parents of the children. Familial experiences were indexed with the parents’ level of education (11 levels, 7.4±1.6, group mean ± SD, range 4.5–10.5) and years of experience with siblings (i.e. the product of age and number of siblings: 4.3±3.4, range 0–15.2; number of siblings: 1.2±0.7, range 0–3). Non-familial experiences were indexed with the time spent at daycare (days per week) between the age of 0 and 4 (mean over these four years; 1.7±0.9 days per week, range 0.25–3). We did not consider between ages 4 and 5 given that in the Netherlands it is customary to start primary school at age 4.

### Data Analysis

Audio- and video-recordings of the participant’s behavior were analyzed offline. Those trials in which the child behavior revealed procedural uncertainties (e.g. failing to return to the nest within 15 seconds, or interrupting the bird movements to look at the location of the acorn in the instruction game board) were excluded, leaving 80.1±13.4% (mean ± SD) of the original trials for further analysis (∼40 trials; four participants interrupted their performance after 30 trials).

This study builds on the findings of a previous report involving the same task and obtained in a group of women [21], showing that the communicator’s belief about age of the addressee changed communicative behavior. More precisely, these adults spent longer time on communicatively relevant locations of the game board when interacting with a presumed child addressee (vs. an adult addressee), i.e. using time as a tool to place emphasis on target information. The first goal of this study was to replicate this finding in a group of five-year-old children. Accordingly, we considered the same dependent variable (namely, Time spent on game board locations), using the same statistical comparison, namely a two-way ANOVA with factors Addressee (Toddler, Child) and Location (Target, Non-target). The Time spent on game board location by the Communicator was calculated as the time interval between the first contact of the finger on the touch screen within the area of a square of the game board (either a Target or a Non-target location) and the subsequent contact of the finger within the area of a neighboring square of the game board. We considered the mean time spent on those location types per trial. It should be emphasized that, given the absence of temporal restrictions on the total time the children could spend on the game board, the time spent on target locations and the time spent on non-target locations could vary independently.

**Figure 2:**
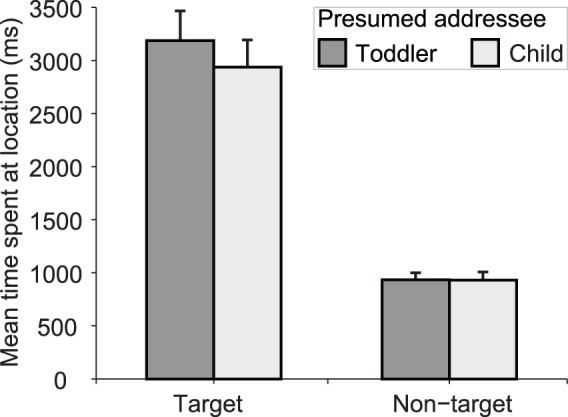
Communicative adjustments. Time spent on Target and Non-target locations (during event 2 in Figure 1B; mean ± SEM; average time per trial) by the participants as a function of presumed Addressee (Toddler, Child).

Having replicated the findings of [21] in this group of five year-olds (Figure 2), we used a multiple linear regression analysis to assess the differential contribution of familial and non-familial sources of social interactions experienced by these children in the first four years of their life. These three independent variables (i.e. parents’ level of education, years of experience with siblings, and time spent at daycare, see above) were jointly considered in the multiple regression analysis, with the degree of communicative adjustment observed in each child as dependent variable (i.e. the relative difference, [toddler – child]/[child], in time spent on Target locations between presumed toddler and child Addressee). This statistical approach allows one to make specific inferences on the inter-subject variance accounted for one variable, over and above the variance accounted by the other variables included in the multiple regression model.

## Results

### Communicative Success

The percentage of successfully communicated trials was 63.4±8.0% (mean ± SD). This is well above chance level (7.7%; 13 potential target locations).

### Communicative Adjustments

We tested whether 5-year-old children are able to adapt their referential communicative behavior (event 2 in Figure 1B) to the presumed age, or cognitive level, of their interlocutor. A two-way analysis of variance revealed a significant interaction of the factors Addressee (Toddler, Child) and Location (Target, Non-target) on the mean time spent on game board locations during the movement epochs, *F*(1,23) = 5.4, *p* = .03. This interaction was driven by the fact that the 5-year-old children spent more time on the Target locations (containing the acorn) when they thought to be interacting with the toddler Addressee as compared to the child Addressee, *t*(23) = 2.6, *p* = .014, two-sided paired *t*-test. There was no difference between the two Addressee types for the mean time spent on the Non-target locations (other visited locations), *t*(23) = 0.04, *p* = .97; see Figure 2.

### Effects of Social Environment

**Figure 3:**
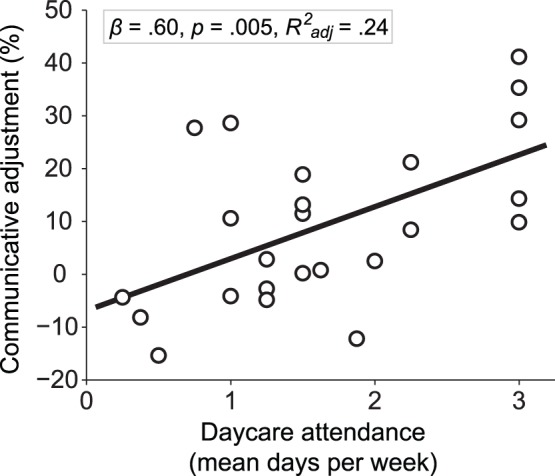
Effect of daycare attendance on communicative adjustments. Individual communicative adjustments of 5-year-old participants plotted against days spent at daycare before starting school (mean of ages 0 to 4). Communicative adjustment was indexed by the relative difference of time spent on Target locations (see Figure 2) between presumed toddler and child Addressees.

We evaluated whether quantitative indexes of developmental exposure to social interactions of the child could explain inter-individual variability in the communicative adjustment observed over the whole group. A multiple linear regression analysis indicated that daycare attendance (i.e. mean days per week spent at daycare before starting school) predicted the communicative adjustments made by the 5-year-old participants, *R^2^* = .34, *F*(3,23) = 3.4, *p* = .039 (full model), *Beta = *.598, *p* = .005, *R^2^_adj_* = .24 (daycare attendance); see Figure 3. Parents’ level of education (*Beta* = −.14, *p* = .45) and years of experience with siblings (*Beta = *.04, *p* = .84) did not significantly account for inter-subject variance in communicative adjustments.

## Discussion

We have tested whether the expression of audience design abilities in 5-year-old children is modulated by their previous history of social interactions. Participants were asked to influence the behavior of an addressee, in an experimental setting where no pre-existing communicative conventions were immediately available. In fact, the communicative means made available to the children were purportedly limited, challenging them to devise new communicative behaviors that could be understood by the addressees. There are three main results. First, 5-year-old children were able to influence the mental states of others even at their first encounter with a novel communicative setting. This communicative behavior was internally generated by the children, and motorically different from the behavior of the two presumed addressees (Figure 1B). Second, the mere belief of communicating with addressees of different ages selectively influenced the communicative behavior of the participants. The children spent longer at communicatively relevant locations when interacting with a presumed toddler addressee as compared to a presumed child addressee. This communicative adjustment was not a generic priming effect, being absent in communicatively irrelevant locations of the game-board. Third, the communicative adjustment observed in the children was predicted by the time spent at daycare during the previous years of their life. This latter finding refines the notion that human communicative skills might be shaped early during development [14], [26], emphasizing the fundamental role of non-familial interactions in the gradual construction of children’s social understanding and abilities to influence the mental states of others [5], [7].

It has been suggested that children gradually construct mental variables through the regularities they experience within social interaction [5], [7], [27]. In contrast to a large body of work focusing on verbal reports of children’s ability to *attribute* mental states to other people, as during Theory of Mind tasks [16], [28], here we considered children’s ability to *influence* the mental states of others through non-verbal behaviors, i.e. the magnitude of their communicative adjustments. These spontaneous adjustments provided a sensitive index for quantifying inter-individual differences in communicative abilities close to the onset of those abilities. This sensitivity might arise from the implicit nature of the index of audience design used in this study, in line with findings previously obtained during language comprehension in children of similar age [29], [30]. Namely, in contrast to previous work exploring how a child’s inhibitory control handles the conflict between the knowledge of the child and that of the addressee [30], in this study we manipulated the presumed abilities of the addressee, minimizing demands on the control abilities of the child [31].

The magnitude of communicative adjustments in 5-year-old children was predicted by the time spent in daycare during previous years of their life, over and above the effects accounted for by measures of the familial social environment (sibling experience, educational level of the parents). One possible mechanism accounting for this observation might relate to the importance that overheard communicative interactions have on the linguistic development of a child [32], [33], [34]. Namely, kindergarten attendance might considerably boost the variety of children’s experience with this source of pragmatic inputs, enhancing their communicative skills. More generally, the structured social interactions afforded by a daycare environment (e.g. cooperative play, frequent integration of new group members) might provide the child with a larger set of communicative challenges than those experienced within a relatively stereotyped familial environment [35]. These challenges might differ substantially from those experienced in a familial environment. In kindergarten, a child needs to communicate with a multitude of agents, and those agents lack a genetic reason for collaboration. Finally, kindergarten provides children with caregiving ‘alloparents’ that might boost their socio-emotional development [36].

It remains to be seen how the present findings, showing stronger effects of non-familial over familial experiences on the development of referential communicative adjustments, can be reconciled with previous reports, showing that measures of familial interactions predicted ‘false belief understanding’ [12], [13], as assessed with verbal reports. One possibility is that the communicative adjustments observed in this study might be mainly driven by children’s assumptions on the presumed cognitive capacities of the addressees, rather than by children’s understanding that the beliefs, desires, or intentions of other agents differ from reality [11], [13]. Differences in outcome measures might also play a role, e.g. implicit measures of knowledge about a communicative interaction (as gathered through eye movements, reaction time, or movement times) vs. explicit verbal reports requiring a degree of executive control [29], [31].

This study opens the way for systematic and sensitive investigations into the contributions of early social experiences towards children’s communicative abilities, raising the possibility to chart the developmental trajectories generated by familial and non-familial social interactions (e.g. siblings, parents, non-sibling peers, alloparents) through longitudinal studies with objective measures of the time spent on those interactions.

## Supporting Information

Figure S1 **Time-variability of the communicative adjustments.** Time spent on Target and Non-target locations by the participants as a function of presumed Addressee (Toddler, Child) and Task epoch (First Half, Second Half).
(EPS)

Table S1 **Explanatory variables and their predictive value on communicative adjustment as determined with single linear regression analyses.**
(DOCX)
