# Supplementary material for: Early Social Experience Predicts Referential Communicative Adjustments in Five-Year-Old Children
Source: PLoS One. 2013 Aug 29;8(8):e72667. doi: 10.1371/journal.pone.0072667 (PMC3757022; doi:10.1371/journal.pone.0072667)
Supplement: Table S1 — Explanatory variables and their predictive value on communicative adjustment as determined with single linear regression analyses. (DOCX) [file pone.0072667.s002.docx]

# Supplemental Material for

Early social experience predicts

referential communicative adjustments

in five-year-old children

Arjen Stolk^a,1^, Sabine Hunnius^a^, Harold Bekkering^a^, Ivan Toni^a^

^a^Radboud University Nijmegen, Donders Institute for Brain, Cognition and Behaviour

**Supplemental Methods**

## *Experimental procedure*

During the experiment, the participant sat in a sound-proof experimental room, facing a monitor displaying the digital game board (Figure 1A), while being video- and audio-recorded. An experimenter sat next to the participant, providing the task instructions and the trial-specific location of the target object (i.e. an acorn), but playing no part in the communicative game. A confederate sat outside the experimental room facing another monitor, showing the same game board seen by the participant. First, each child was familiarized with the experimental setup (5 trials, ~ 5 min). During these familiarization trials the child was encouraged to freely move the bird around the game board, experiencing the constraints on the bird’s movements as described in the experimental design. The child was also instructed to return the character to the nest (center game board location) at the end of his movements. This task requirement was emphasized by a continuous sound (a bird squeak) that started when the child moved away from the nest and stopped when the child returned the bird to the nest.

Second, each child was informed that he would be playing an interactive game with two addressees in turns; either a toddler (‘2-year-old’) or a same–age peer (‘5-year-old’). They were told that the game partners were sitting in other rooms and that they could see the bird token and the digital game board on their monitors. In fact, the confederate performed the role of both addressees, while remaining blind to which one of the two roles he was performing in any given trial. At the onset of each trial the confederate did not know the location of the acorn, and he could infer that location only from the movements of the participant. There were a total of 50 trials (~35 min, Figure 1C). The participant alternately interacted with the two pseudo addressees in blocks of five trials. There were two pairs of fictitious child-toddler addressees, two presentation orders of child-toddler addressees, and two sets of target configurations, counterbalanced over participants. The game was programmed using Presentation (Neurobehavioral Systems, Albany, CA, USA) software on a Windows XP personal computer.

***Manipulation of task difficulty***

Task difficulty was manipulated by introducing a disparity between the movements allowed to the bird token (i.e. translations to the center of each square) and the potential location of target object (i.e. the white circles). Namely, the bird could not be overlaid on the precise location of the acorn when a square contained more than one white circle. ‘Easy, medium, and hard trials’ are distinguished according to their local characteristics, i.e. trials where the acorn was located in a square with one, two, or three white circles, respectively. Importantly, even in relatively easy trials the child needed to find a way to make clear to the Addressee which square contained the acorn, disambiguating it from other squares on the game board, in particular from those squares visited by the bird while moving across the game board. Easy, medium, and hard trials were pseudo-randomly intermixed such that there was an overall increase in difficulty during the course of the experiment. The rationale for manipulating task difficulty was to challenge the participants toward the creation of new communicative behaviors.

The percentage of successfully communicated *easy* trials was 94.6 ± 6.7%. *Medium* and *hard* trials were successfully communicated on 57.1 ± 11.7% and 16.8 ± 19.9% of the trials, respectively.

Note that the above mentioned classification scheme is not suitable for objectively and consistently categorizing the communicative difficulty experienced by the participants, since trials with similar local characteristics might actually differ in difficulty. For instance, the top right square of the game board contains the same number of potential target locations (i.e. 2 circles, see Figure 1A) as the square in the middle of the bottom row. However, discriminating between the two circles in the latter square may involve different communicative strategies than discriminating between the two circles in the top right square: The left circle in the bottom middle square may require a detour over other squares to disambiguate it from the top circle in the same square. Besides the number of potential target locations, the exact spatial configuration of those target locations may lead to different communicative strategies and difficulties.

**Supplemental Analyses**

In the following sections, we report a number of additional, post-hoc analyses designed to verify the specificity of the findings reported in the main text.

***Communicative specificity of the adjustments in children behavior***

We performed additional analyses to verify whether the adjustments reported in the main analysis (Figure 2) were specific to communicative aspects of the children’s behavior. The main analysis already addresses this issue by contrasting the time spent on a communicatively relevant location of the game board (i.e. Target location) with the time spent on other locations of the game board (Non-target locations). Here we report the results of additional post-hoc comparisons on dependent variables selected for being closer to motoric than communicative aspects of the children’s performance. The aim of these comparisons was to assess whether the interaction effect found in the Addressee * Location ANOVA could in fact be a generic motoric effect evoked by the belief of communicating with a Child or a Toddler. We considered the dependent variables of planning times (duration of event 1, Figure 1b), movement times (duration of event 2), the number of moves made by the participants, movement times of the confederate (duration of event 3), and communicative success. These additional post-hoc tests were calculated for each of the two Addressee types (Toddler, Child) and statistically compared by means of two-sided paired *t*-tests. It should be emphasized that these post-hoc tests dealt exclusively with one of the main effects considered in the two-way ANOVA (namely, Child vs. Toddler difference), and therefore those tests could not increase the risk of finding a false positive interaction in the two-way ANOVA.

We found that the dependent variables of overall planning and movement times (*p* = .13 and *p* = .17) and the number of moves made by the participants (*p* = .87), as well as performance and response times of the confederate (*p* = .12 and *p* = .10), were matched between both Addressee types. These results support the notion that the adjustments reported in the main analysis (Figure 2) were specific to communicative aspects of the children’s behavior.

***Temporal dynamics of the communicative adjustments***

We performed an additional analysis to test whether the children’s communicative adjustments reported in the main analysis (Figure 2) were present from their first encounter with this communicative setting. The two-way ANOVA with factors Addressee and Location reported in the main text (Figure 2) was therefore further qualified by separately testing this interaction in the first and in the second half of the experiment, through a three-way ANOVA with factors Addressee, Location, and Task epoch. There was a significant three-way interaction, *F*(1,23) = 5.2, *p* = .033, indicating that the interaction effect of Addressee and Location on the Time spent on game board locations was present in the first half of the experiment, *F*(1,23) = 5.9, *p* = .024, but not in the second half, *F*(1,23) = 0.4, *p* = .56; see Figure S1. These results indicate that the communicative adjustments reported in the main analysis (Figure 2) were present from the children’s first encounter with this communicative setting. In fact, the communicative adjustments were attenuated with experience of the ongoing communicative interaction, in line with the findings of (Newman-Norlund, et al., 2009).

Insert *Figure S1* about here

***Specificity of the social environmental parameters considered in this study***

We performed additional post-hoc analyses to verify the stability and specificity of the predictive relation between daycare attendance and communicative adjustments reported in the main analysis (Figure 3). We assessed the predictive value of a number of additional independent variables (Table S1) on the magnitude of the children’s communicative adjustments. These variables capture general measures of task performance, children’s experience with audio-visual devices, and derivative measures of the main sources of social interactions experienced by the five-year-olds (i.e. familial and non-familial). We used single linear regression analyses to maximize the sensitivity of these control analyses, at the costs of reduced specificity, in order to assess whether the result obtained in the multiple regression analysis (Figure 3) could in fact be derived from other parameters describing the children’s environment or their task performance. Even under these statistically lenient conditions, these alternative parameters were not accounting for significant portions of the children’s communicative adjustments. These results support the notion that the predictive value of daycare attendance on communicative adjustments reported in the main analysis (Figure 3) was specific across a number of conceivable parameters.

Insert *Table S1* about here

**Table S1.** Explanatory variables and their predictive value on communicative adjustment as determined with single linear regression analyses.

|  | *Beta* | *t-statistic* | *significance* | *R^2^_adj_* |
| --- | --- | --- | --- | --- |
| *Siblings* |  |  |  |  |
| Number of siblings | -.140 | -0.662 | .515 | -.029 |
| Number of younger siblings | -.125 | -0.125 | .589 | -.029 |
| Number of older siblings | -.011 | -0.053 | .958 | -.045 |
| Cumulated sibling age | .126 | 0.594 | .559 | -.029 |
| Birth order (e.g. first born) | -.011 | -0.053 | .958 | -.045 |
| *Socio-economic status* |  |  |  |  |
| Mother’s education level | .052 | 0.243 | .811 | -.043 |
| Father’s education level | -.077 | -0.362 | .721 | -.039 |
| *Task performance* |  |  |  |  |
| Communicative success | -.163 | -0.774 | .447 | -.018 |
| Relative communicative success | .032 | 0.152 | .881 | -.044 |
| Communicative success easy trials | -.049 | -0.228 | .822 | -.043 |
| Communicative success medium trials | .347 | 1.738 | .096 | .081 |
| Communicative success hard trials | -.282 | -1.379 | .182 | .038 |
| *Other* |  |  |  |  |
| Time spent at home with computer and television | .231 | 1.112 | .278 | .010 |
